# Ultraslow light realization using an interacting Bose–Einstein condensate trapped in a shallow optical lattice

**DOI:** 10.1038/s41598-022-08250-9

**Published:** 2022-03-15

**Authors:** H. Mikaeili, A. Dalafi, M. Ghanaatshoar, B. Askari

**Affiliations:** 1 Laser and Plasma Research Institute, Shahid Beheshti University, Tehran, Iran; 2 Department of Physics, Shahid Beheshti University, Tehran, Iran

**Keywords:** Matter waves and particle beams, Quantum physics, Quantum metrology

## Abstract

In this article, we propose an experimentally feasible scheme for the ultraslow light realization based on the optomechanically induced transparency (OMIT) phenomenon using a hybrid optomechanical system consisting of a one-dimensional Bose–Einstein condensate trapped in a shallow optical lattice considering the nonlinear effect of atom-atom interaction. It is shown how the system can switch from the normal mode splitting to the OMIT regime by manipulation of the *s*-wave scattering frequency of atomic collisions when the cavity is pumped at a fixed rate. Then, it is shown that an ultraslow light with a time delay more than 150 ms corresponding to a group velocity about 1 mm/s is achievable by controlling the optical lattice depth as well as the strength of atom-atom interaction and the number of atoms. Importantly, such an ultraslow light is detectable in the output of the cavity since it occurs in the frequency region of coupling-probe detuning where the reflection coefficient of the cavity is maximum.

## Introduction

The phenomenon of electromagnetically induced transparency (EIT), which was first observed in atomic vapors by Harris and coworkers^1–3^, has attracted much attention of the physics community over the last few decades. One of the most fascinating features of EIT has been the realization of slow light^4^ where the group velocity of light becomes much lower than that in the vacuum and has important applications in optical telecommunication and interferometry^5,6^. More recently, it has been shown that optomechanical cavities with moving mirrors can be used as another platform for manifestation of EIT which is called optomechanically induced transparency (OMIT)^7–18^.

In a bare optomechanical system (OMS), where the moving end mirror or the membrane in the middle acts as a mechanical oscillator, the radiation pressure of the cavity optical field is coupled with the vibrational mode of the mechanical oscillator through the optomechanical coupling^19–21^. In parallel, there are hybrid optomechanical systems which consist of cavities containing atomic systems like an ensemble of ultracold atoms or a Bose–Einstein condensate (BEC) where the collective mode excitation of the atomic field plays the role of the mechanical oscillator which couples to the radiation pressure of the cavity^22–24^. One of the most interesting features of such hybrid systems containing BEC is the nonlinear effect of the atom-atom interaction which plays the role of an atomic parametric amplifier^25–32^. It has been shown that the nonlinear effect of atomic collisions may be used for the generation of Casimir photons^33^, strong quadrature squeezing^34^, and ultraprecision quantum sensing^35–37^ .

In recent years, optomechanical systems have been proposed as suitable candidates to substitute for atomic gases in order to generate slow and fast light based on the phenomenon of OMIT^38–40^. Besides, it has been shown that hybrid optomechanical systems consisting of atomic systems or BEC may provide more controllability in fast and slow light realization^41–47^. Furthermore, the phenomenon of Fano resonance which was observed for the first time in some of the Rydberg spectral atomic lines, can be also observed in optomechanical systems^48–50^.

In the study of ultracold atoms or BECs trapped inside periodic potentials of optical lattices, there are two important regimes of deep and shallow optical lattice where the system is described based on different mathematical models^51^. In the regime of deep optical lattice, which is also referred to as the tight-binding limit, the system is described by the Bose–Hubbard model^51^ where the atomic field is expanded in terms of the Wannier wave functions. It has been shown^52^ that in a system consisting of a BEC inside a deep optical lattice which obeys the Bose-Hubbard model, the time delay of the slow light group velocity can be increased up to about $55\,\upmu\mathrm{s}$ in the best conditions by controlling the effective cavity detuning, the pumping rate of the cavity, and the strength of atomic collisions. On the other hand, for obtaining slow lights with longer time delays, it is necessary to decrease the depth of the optical lattice by decreasing the pumping rate of the cavity. However, in the limit of shallow optical lattice, the Bose-Hubbard model loses its validity and the system should be described by a model in which the atomic wave function is expanded in terms of plane waves and can be considered as a single-mode quantum field in the Bogoliubov approximation^53,54^. It has been shown^55^ that in a hybrid optomechanical system consisting of a BEC trapped in a shallow optical lattice, the time delay of the slow light can be enhanced up to 0.8 ms by decreasing the depth of the optical lattice in the absence of atom-atom interaction.

Motivated by the above-mentioned investigations on slow light realization, in this article we propose an experimentally feasible scheme for the ultraslow light realization based on the OMIT phenomenon using a hybrid optomechanical system consisting of a one-dimensional BEC trapped in a shallow optical lattice considering the nonlinear effect of atom-atom interaction. It is assumed that the cavity is pumped by a coupling laser, which is responsible for the generation of the optical lattice and is tuned at the red sideband of the cavity effective frequency, and simultaneously is probed by another laser with a much weaker pumping rate. It is shown that at a fixed value of the coupling laser pumping rate, one can make the system change its regime from the normal mode splitting (NMS) to the OMIT by increasing the *s*-wave scattering frequency of atomic collisions. Then, we show that an ultraslow light with a time delay more than 150 ms, which corresponds to a group velocity as low as 1 mm/s, is achievable by decreasing the optical lattice depth through decreasing the pumping rate of the cavity and also by increasing the strength of atom-atom interaction through the transverse trapping frequency of the BEC and controlling the number of the atoms of the BEC. We also explicitly show that the mentioned time delay occurs in the frequency region of coupling-probe detuning where the reflection coefficient of the cavity is maximum and therefore an ultraslow light with a considerable amplitude is physically observable in the output field of the cavity.

The paper has been organized as follows: In “System Hamiltonian” section the Hamiltonian of the system is described and in “Dynamics of the system” section the dynamics of the system is modeled based on the Heisenberg-Langevin equations. In “Results and discussion” section it is shown how an ultraslow light with a considerable time delay can be realized in the output of the cavity based on the OMIT phenomenon. Finally, the summary and conclusions are given in “Summary and conclusions” section.

## System Hamiltonian

**Figure 1:**
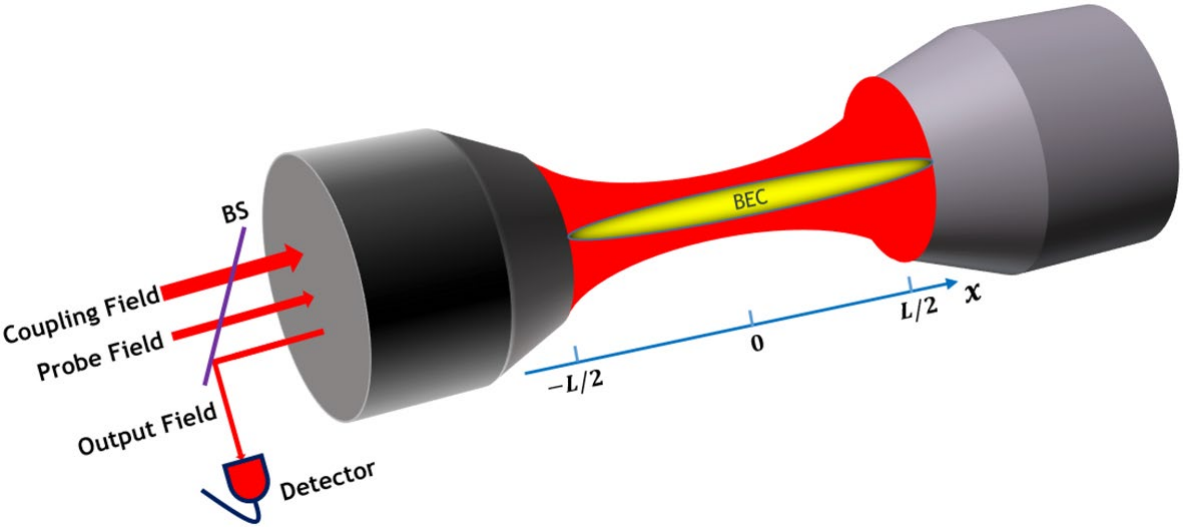
A Fabry-Perot cavity containing a BEC is simultaneously driven from the left mirror and along the cavity axis by a coupling field at rate $\varepsilon _c$ and a weak probe field at rate $\varepsilon _p$. An ultraslow light can be detected on the left hand side of the cavity.

The system consists of a Fabry-Perot cavity with length *L* and resonance frequency $\omega _{0}$ containing a Bose-Einstein condensate (BEC) as has been depicted in Fig. 1. The cavity is driven simultaneously by two external lasers through the partially transparent left mirror and along the cavity axis. The strong coupling field with frequency $\omega _{c}$ and pumping rate $|\varepsilon _c|=\sqrt{2\kappa _e P_c/\hbar \omega _c}$ generates an optical lattice which interacts with the so-called mechanical mode of the BEC through a radiation pressure interaction while the weak probe field with frequency $\omega _{p}$ and pumping rate $|\varepsilon _p|=\sqrt{2\kappa _e P_p/\hbar \omega _p}$ probes the system response. Here, $P_c$ and $P_p$ are, respectively, the powers of the coupling and probe lasers and $\kappa _{e}$ denotes the external decay rate of the cavity.

The matter quantum field of the BEC, which consists of *N* identical two-level atoms with mass $m_{a}$ and transition frequency $\omega _{a}$, interacts with the single mode of the cavity optical lattice in the dispersive regime, where the coupling laser frequency is far detuned from the atomic resonance so that $\Delta _{a}=\omega _{c}-\omega _{a}\gg \Gamma _{a}$ with $\Gamma _{a}$ being the atomic linewidth. In this way, transition to the upper level is negligible, and consequently spontaneous emission is unlikely to happen^56–58^. Under the above-mentioned conditions, the Hamiltonian of the system in the second quantization formalism can be written as

1 $$\begin{aligned} {\hat{H}}= & {} \hbar \omega _{0} {\hat{a}}^{\dagger }{\hat{a}}+\int _{-L/2}^{L/2} dx {{\hat{\Psi }}}^{\dagger }(x)\left(\frac{-\hbar ^2}{2m_a}\frac{d^2}{dx^2} + \hbar U_0 \cos ^2(kx) {\hat{a}}^{\dagger }{\hat{a}}+\frac{1}{2} U_{s}{{\hat{\Psi }}}^{\dagger }(x){{\hat{\Psi }}}(x)\right ){{\hat{\Psi }}}(x)\nonumber \\&+i\hbar \left(\varepsilon _c e^{-i\omega _c t}{\hat{a}}^{\dagger } - \varepsilon _c^{*} e^{i\omega _c t}{\hat{a}}\right )+i \hbar \left(\varepsilon _p e^{-i\omega _p t}{\hat{a}}^{\dagger } - \varepsilon _p^{*} e^{i\omega _p t}{\hat{a}}\right), \end{aligned}$$

where ${{\hat{\Psi }}}(x)$ is the annihilation operator of the atomic field in position space and ${\hat{a}}$ is the annihilation operator of the cavity mode in momentum space. In addition, $k=\omega _c/c$ is the optical lattice wave number, $U_0 =g_0^2/\Delta _a$ is the depth of optical lattice potential per single-photon inside the cavity, $g_0$ is the vacuum Rabi frequency, and $U_s =4\pi \hbar ^2 a_s/m_a$ is the atom–atom scattering strength with $a_s$ being the two-body s-wave scattering length. It should be noted that in the second quantization formalism, the electromagnetic field (*E*(*x*, *t*), *B*(*x*, *t*)) inside the cavity can also be expanded as a Fourier series in terms of the cavity normal modes in the momentum space^59^. Since the cavity is pumped by the coupling laser whose frequency is near to one of the resonance frequencies of the cavity, a single mode of the cavity can be excited if the laser linewidth is much smaller than the frequency distance between the successive optical modes. In this way, one can achieve a single-mode optical field inside the cavity whose Hamiltonian is given by the first term of Eq. (1).

On the other hand, the Hamiltonian of the BEC and its interaction with the single mode of the optical field is given by the second term of Eq. (1). As has been investigated in Refs.^53,54^, the BEC has several bands of energy where each of them has a lot of atomic modes so that the atomic field operator should be expanded in terms of them as the following equation

2 $$\begin{aligned} {{\hat{\Psi }}}(x)=\frac{1}{\sqrt{L}}\sum _{n}\sum _{m=-l/2}^{l/2}{\hat{b}}_{nm} e^{i(n+m/l)2kx}, \end{aligned}$$

where *n* is the Bloch band index and *m* corresponds to quasi-momentum number with $l=kL/\pi$. In the regime of weak optical lattice, where the condition $U_0 \langle a^{\dagger }a\rangle \le 10\omega _R$ is satisfied ($\omega _R=\hbar k^2/2m_a$ is the recoil frequency of atoms), the above expansion can be restricted to the lowest band numbers $n=0,\pm 1$. Besides, if the system starts from a homogeneous BEC, only the cosine parts of the exponential functions are excited because of the parity conservation so that the atomic field can be expanded in terms of new operators ${\hat{c}}_{nm}=({\hat{b}}_{nm}+{\hat{b}}_{-n,-m})/\sqrt{2}$ where under the above-mentioned conditions the atomic field reduces to the following two-mode quantum field

3 $$\begin{aligned} {{\hat{\Psi }}}(x)=\frac{1}{\sqrt{L}}{\hat{c}}_{00}+\sqrt{\frac{2}{L}}{\hat{c}}_{01} \cos (2kx). \end{aligned}$$

In the regime of weak optical lattice and for large *N* the depletion of the initial condensate remains weak so that one can use the Bogoliubov approximation^58^ and treat the zero-momentum mode classically, i.e., ${\hat{c}}_{00}\approx \sqrt{N}$. In this way, the atomic field annihilation operator of the BEC in position space can be expanded as the following single-mode quantum field

4 $$\begin{aligned} {{\hat{\Psi }}}(x) = \sqrt{\frac{N}{L}} + \sqrt{\frac{2}{L}}\cos (2kx) \hat{c}, \end{aligned}$$

where $\hat{c}\equiv {\hat{c}}_{01}$ is the annihilation operator of the first excited mode of the BEC (against the lowest mode ${\hat{c}}_{00}$) in momentum space which obeys the commutation relation $[\hat{c},\hat{c}^{\dagger }]=1$.

We also define the position and momentum quadratures of the BEC mode as $\hat{Q}=(\hat{c}+\hat{c}^{\dagger })/\sqrt{2}$ and $\hat{P}=(\hat{c}-\hat{c}^{\dagger })/\sqrt{2}i$ which satisfy the commutation relation $[\hat{Q},\hat{P}]=i$ according to the commutation relation between $\hat{c}$ and $\hat{c}^{\dagger }$. Now, by substituting Eq. (4) into Eq. (1) and performing some calculations, the Hamiltonian of the system in the frame rotating at the coupling field frequency is obtained as the following form in terms of the position and momentum quadratures of the BEC

5 $$\begin{aligned} \hat{H} = \hbar \delta _{c} \hat{a}^{\dagger }\hat{a}+ \hbar \zeta \hat{a}^{\dagger }\hat{a} \hat{Q} + \frac{1}{2} \hbar \Omega _{c}(\hat{P}^2+\hat{Q}^2) +\frac{1}{2} \hbar \omega _{sw} \hat{Q}^2 + i \hbar (\varepsilon _c \hat{a}^{\dagger } - \varepsilon _c^{*} \hat{a}) + i \hbar (\varepsilon _p e^{-i\delta t} \hat{a}^{\dagger } - \varepsilon _p^{*} e^{i\delta t} \hat{a}), \end{aligned}$$

where $\delta _{c}=\Delta _c + \frac{1}{2} N U_0$ is the Stark-shifted cavity frequency due to the presence of the BEC, $\Delta _{c}= \omega _0 - \omega _{c}$ is the detuning between the cavity resonance and coupling laser frequency, $\zeta =\frac{1}{2}\sqrt{N}U_0$ is the optomechanical coupling between the so-called mechanical mode of the BEC and the optical field of the cavity, $\Omega _{c}= 4 \omega _{R} + \omega _{sw}/2$ with $\omega _{sw}=8\pi \hbar a_{s}N/m_{a}Lw^2$ being the *s*-wave scattering frequency of the atomic collisions (*w* is the waist radius of the optical mode), and $\delta =\omega _p-\omega _{c}$ is the coupling-probe detuning. Based on the Hamiltonian of Eq. (5), the system behaves effectively as an optomechanical system where the Bogoliubov mode of the BEC plays the role of the mechanical mode of a moving mirror which is coupled with the cavity optical lattice through a radiation pressure interaction (the second term in Eq. (5)) with the difference that in the present model there is another nonlinear term which originates from the atom-atom interaction (the fourth term in Eq. (5)). It should be emphasized that the concept of the position and momentum operators of the Bogoliubov mode of the BEC is different from that of a moving mirror of a bare OMS. Nevertheless, the present system is an analog of a standard OMS with a moving mirror.

## Dynamics of the system

The dynamics of the system is described by the following Heisenberg-Langevin equations of motion deduced from the Hamiltonian of Eq. (5):

6a $$\begin{aligned} \frac{d \hat{a}}{dt}= & {} -(\kappa + i\delta _{c}){\hat{a}} - \zeta \hat{Q}\hat{a} + \varepsilon _c + \varepsilon _p e^{-i\delta t} + \sqrt{2\kappa _e}{\hat{a}}_{in}+ \sqrt{2\kappa _i}{\hat{a}}_{int}, \end{aligned}$$

6b $$\begin{aligned} \frac{d\hat{P}}{dt}= & {} -\gamma _B \hat{P} -(\Omega _{c}+\omega _{sw})\hat{Q} -\zeta \hat{a}^{\dagger }\hat{a} + {\hat{P}}_{in}, \end{aligned}$$

6c $$\begin{aligned} \frac{d\hat{Q}}{dt}= & {} \Omega _c \hat{P}, \end{aligned}$$

where $\sqrt{2\kappa _e}{\hat{a}}_{in}$ is the input noise originated from the input–output coupling while $\sqrt{2\kappa _i}{\hat{a}}_{int}$ is the internal noise due to non-perfect mirrors or scattered light from the residue air molecules in the cavity with $\kappa _i$ being the internal decay rate of the cavity. In fact, the intra-cavity field leaks through the left mirror at the rate of $\kappa _e$ which can be detected as the output field of the cavity and also is dissipated by any inaccessible channels at the rate of $\kappa _i$ so that the damping rate of the cavity field amplitude is $\kappa =\kappa _{e}+\kappa _i$^60^. Furthermore, the coupling parameter $r_c=\kappa _e/\kappa$ is defined to describe the output coupling ratio^60^. Besides, $\gamma _B$ is the damping rate of the Bogoliubov mode of the BEC and ${\hat{P}}_{in}$ is the input noise of the BEC atomic field with zero mean value.

Using the mean-field approximation, i.e., $\langle \hat{a}\hat{b}\rangle = \langle \hat{a} \rangle \langle \hat{b} \rangle$, which means that the two operators are assumed to be uncorrelated and considering $\langle {\hat{a}}_{in}\rangle =\langle {\hat{a}}_{int}\rangle =\langle {\hat{P}}_{in}\rangle =0$^7,45^, the equations of motion for the mean values of the operators are obtained from Eqs. (6a–6c) in the following form

7a $$\begin{aligned} \frac{d \langle \hat{a}\rangle }{dt}= & {} -(\kappa + i\delta _{c})\langle {\hat{a}}\rangle - \zeta \langle \hat{Q}\rangle \langle \hat{a}\rangle + \varepsilon _c + \varepsilon _p e^{-i\delta t}, \end{aligned}$$

7b $$\begin{aligned} \frac{d\langle \hat{P}\rangle }{dt}= & {} -\gamma _B \langle \hat{P}\rangle -(\Omega _{c}+\omega _{sw})\langle \hat{Q}\rangle -\zeta \langle \hat{a}^{\dagger }\rangle \langle \hat{a}\rangle , \end{aligned}$$

7c $$\begin{aligned} \frac{d\langle \hat{Q}\rangle }{dt}= & {} \Omega _c \langle \hat{P}\rangle . \end{aligned}$$

By eliminating $\langle \hat{P}\rangle$ from Eqs. (7b) and (7c) the dynamical equation of the Bogoliubov mode of the BEC can be rewritten as the following second order differential equation

8 $$\begin{aligned} \frac{d^2\langle \hat{Q}\rangle }{dt^2} +\gamma _B\frac{d\langle \hat{Q}\rangle }{dt}+ \omega _B^2 \langle \hat{Q}\rangle = -\Omega _{c}\zeta \langle \hat{a}^{\dagger }\rangle \langle \hat{a}\rangle , \end{aligned}$$

which is the dynamical equation of a driven-damped simple harmonic oscillator with the effective resonance frequency $\omega _B=\sqrt{(4\omega _{R}+\frac{1}{2}\omega _{sw})(4\omega _{R}+\frac{3}{2}\omega _{sw})}$, the so-called Bogoliubov frequency. As is evident from Eq. (8) the position quadrature of the Bogoliubov mode is driven by the cavity radiation pressure (the term on the right hand side) and therefore is coupled with Eq. (7a). When the amplitude of the probe field is much weaker than that of the coupling field, the steady-state solutions to the equations of motion, i.e., Eqs. (7a) and (8), to the first order of $\varepsilon _p$ in the rotating frame at frequency $\omega _c$ can be written as:

9a $$\begin{aligned} \langle \hat{a} \rangle= & {} a_0 + a_{+} e^{-i\delta t} + a_{-} e^{i\delta t}, \end{aligned}$$

9b $$\begin{aligned} \langle \hat{Q} \rangle= & {} Q_0 + Q_{+} e^{-i\delta t} + Q_{-} e^{i\delta t}. \end{aligned}$$

According to Eqs. (9a), (9b), the expectation values contain three components. The first components i.e. $a_0$ and $Q_0$, are steady-state solutions at zero order of $\varepsilon _p$ which oscillate at the coupling field frequency $\omega _c$ in the laboratory frame. The second and third components are steady-state solutions at the first order of $\varepsilon _p$ which oscillate, respectively, at the probe frequency $\omega _p$ and four-wave mixing frequency $2\omega _c-\omega _p$ in the laboratory frame^61^.

By substituting Eqs. (9a) and (9b) in the equations of motion, i.e., Eqs. (7a) and (8), the zero order components are obtained as

10a $$\begin{aligned} a_0= & {} \frac{\varepsilon _c}{\kappa + i\Delta }, \end{aligned}$$

10b $$\begin{aligned} Q_0= & {} \frac{-\Omega _c\zeta |a_0|^2}{\omega _B^2}, \end{aligned}$$

where $\Delta = \delta _{c} + \zeta Q_0$ is the effective cavity detuning. On the other hand, by equating the first order terms in either side of the equations of motion with the same frequency, the following set of algebraic equations is obtained

11a $$\begin{aligned} \left( i(\Delta -\delta )+\kappa \right) a_{+}= & {} -i\zeta a_0 Q_{+} + \varepsilon _p, \end{aligned}$$

11b $$\begin{aligned} \left( i(\Delta +\delta )+\kappa \right) a_{-}= & {} -i\zeta a_0 Q_{-}, \end{aligned}$$

11c $$\begin{aligned} \left( \omega _B^2-\delta ^2+i\gamma _B\delta \right) Q_{+}= & {} -\Omega _c \zeta \left( a_0^{*} a_{+} + a_0 a_{-}^*\right) , \end{aligned}$$

11d $$\begin{aligned} \left( \omega _B^2-\delta ^2-i\gamma _B\delta \right) Q_{-}= & {} -\Omega _c \zeta \left( a_0 a_{+}^{*} + a_0^{*} a_{-}\right) . \end{aligned}$$

If $a_0$ is assumed to be real, the unknown coefficients can be obtained by solving Eqs. (11a–11d) as

12a $$\begin{aligned} a_+= & {} \frac{1+if}{i(\Delta -\delta )+\kappa -2f\Delta }\varepsilon _p, \end{aligned}$$

12b $$\begin{aligned} a_{-}= & {} -\frac{a_+^*}{1+i/f^*}, \end{aligned}$$

12c $$\begin{aligned} Q_{-}= & {} Q_{+}^{*}, \end{aligned}$$

where the parameter *f* and the so-called mechanical susceptibility $\chi$ of the Bogoliubov mode of the BEC have been defined as

13a $$\begin{aligned} f= & {} \frac{\chi \zeta ^2 a_0^2}{-i(\Delta +\delta )+\kappa }, \end{aligned}$$

13b $$\begin{aligned} \chi= & {} \frac{\Omega _c}{\omega _B^2-\delta ^2-i\gamma _B \delta }. \end{aligned}$$

To obtain the amplitude of the cavity output (reflected) field at the probe frequency, we use the input-output relation^59^

14 $$\begin{aligned} \varepsilon _{out} + \varepsilon _{in} = 2\kappa _e \langle \hat{a}\rangle , \end{aligned}$$

where $\varepsilon _{in}=\varepsilon _c+\varepsilon _p e^{-i\delta t}$ and $\varepsilon _{out}$ are, respectively, the amplitudes of the input and output fields of the cavity. Similar to Eq. (9a), the cavity output field in the rotating frame with the coupling field frequency can be expanded as

15 $$\begin{aligned} \varepsilon _{out} = \varepsilon _{out0} + \varepsilon _{out+} e^{-i\delta t} + \varepsilon _{out-} e^{i\delta t}. \end{aligned}$$

Here, $\varepsilon _{out0}$, $\varepsilon _{out+}$, and $\varepsilon _{out-}$ are, respectively, the system responses at the coupling field frequency $\omega _c$ , at the probe field frequency $\omega _p$ and at the four-wave mixing frequency $2\omega _c-\omega _p$ in the laboratory frame. By substituting $\langle \hat{a}\rangle$ and $\varepsilon _{out}$ from Eqs. (9a) and (15) and $\varepsilon _{in}$ in the input–output relation of Eq. (14), the system responses can be obtained as

16a $$\begin{aligned} \varepsilon _{out0}= & {} 2\kappa _e a_{0}-\varepsilon _c, \end{aligned}$$

16b $$\begin{aligned} \varepsilon _{out+}= & {} 2\kappa _e a_{+}-\varepsilon _p, \end{aligned}$$

16c $$\begin{aligned} \varepsilon _{out-}= & {} 2\kappa _e a_{-}. \end{aligned}$$

On the other hand, in the critical regime, where the internal resonator loss $(\kappa _i)$ and input-output coupling rate $(\kappa _e)$ are equal so that the coupling parameter becomes $r_c=1/2$, the reflected field amplitude at the probe frequency which is defined as the ratio of output response at the probe frequency ($\varepsilon _{out+}$) to the input probe amplitude , i.e., $\varepsilon _{R} = \varepsilon _{out+}/\varepsilon _p$, is obtained as the following function^8,62^

17 $$\begin{aligned} \varepsilon _{R} = \frac{\kappa (1+if)}{i(\Delta -\delta )+\kappa - 2f \Delta }-1. \end{aligned}$$

Since the total output field at the probe frequency is a complex variable, which can be written as $\varepsilon _R = |\varepsilon _R|e^{i\phi }$ with $\phi$ being its phase, the group velocity delay of the probe field due to the rapid phase variations in the vicinity of the resonance frequency arising from OMIT is given by^14,38,39^

18 $$\begin{aligned} \tau _g = \frac{d\phi (\omega _p)}{d\omega _p}. \end{aligned}$$

## Results and discussion

We investigate the behavior of the real and imaginary parts of the reflected field amplitude of Eq. (17) representing, respectively, the absorption and dispersion as well as the group velocity delay given by Eq. (18) in terms of the coupling-probe detuning $\delta =\omega _p-\omega _c$. For this purpose, we analyze our results based on the experimentally feasible parameters given in Refs.^23,24^. We consider a cavity with length $L=178 \mu$m, damping rate of $\kappa =10^5$ Hz, and bare frequency $\omega _{0}=2.41494\times 10^{15}$ Hz corresponding to a wavelength of $\lambda =780$ nm which contains $N=10^5$ Rb atoms with a transition frequency of $\omega _{a}=2.41419\times 10^{15}$ Hz. The atomic field of the BEC couples with the optical field of the cavity with the atom-field coupling strength $g_{0}=2\pi \times 14.1$ MHz. The recoil frequency of the atoms is $\omega _{R}=23.7$ KHz and the damping rate of the Bogoliubov mode of the BEC is $\gamma =10^{-4}\kappa$.

In the following, we will obtain our results in the red detuned regime of $\Delta =\omega _B$ which leads to the following third order algebraic equation in terms of the coupling laser frequency

19 $$\begin{aligned} (\omega _c - \omega _a)^2 (\omega _0-\omega _c-\omega _B)+\frac{1}{2}N g_{0}^{2}(\omega _c-\omega _a)-\frac{N g_{0}^{4}\Omega _c}{4\omega _{B}^{2}}|a_0|^2=0, \end{aligned}$$

where for a fixed value of $\omega _{sw}$ the value of the Bogoliubov frequency $\omega _B$ is fixed and also the zero-order optical mean-field becomes $|a_0|^2=|\varepsilon _c|^2/(\kappa ^2+\omega _{B}^2)$ based on Eq. (10a). Therefore, there will be three roots for $\omega _c$ for any fixed value of $\omega _{sw}$ where for just one of them the system is stable based on the Routh–Hurwitz criteria^63^.

**Figure 2:**
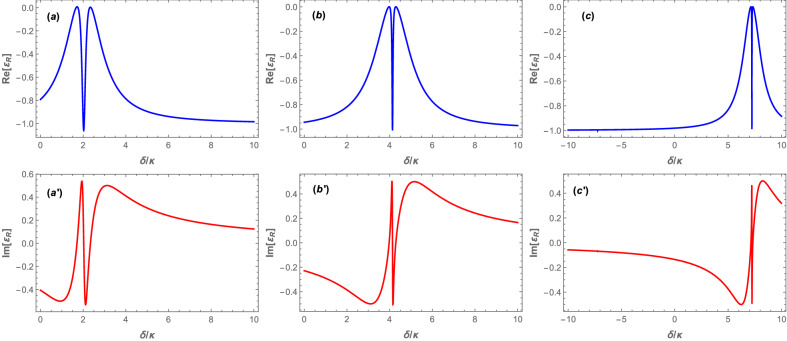
(**a**–**c**) The real and $(\mathrm{a}^{\prime },\mathrm{c}^{\prime })$ imaginary parts of the reflected field amplitude versus the normalized frequency detuning $\delta /\kappa$ for three different values of the *s*-wave scattering frequency: $(\mathrm{a},\mathrm{a}^{\prime })$ $\omega _{sw}=5\omega _R$, $(\mathrm{b},\mathrm{b}^{\prime })$ $\omega _{sw}=15\omega _R$, and $(\mathrm{c},\mathrm{c}^{\prime })$ $\omega _{sw}=30\omega _R$, in the red detuning regime of $\Delta =\omega _B$ when the coupling laser pumping rate is fixed at $|\varepsilon _c| = 0.07\kappa$.

In Fig. 2 we have plotted both the real part of the reflected field amplitude of Eq. (17) expressing the absorption (Fig. 2a–2c), and the imaginary part of the reflected field amplitude expressing the dispersion (Fig. 2$\mathrm{a}^{\prime }$–$\mathrm{c}^{\prime }$), versus the normalized detuning $\delta /\kappa$ for three different values of the *s*-wave scattering frequency: $(\mathrm{a},\mathrm{a}^{\prime })$ $\omega _{sw}=5\omega _R$, $(\mathrm{b},\mathrm{b}^{\prime })$ $\omega _{sw}=15\omega _R$, $(\mathrm{c},\mathrm{c}^{\prime })$ $\omega _{sw}=30\omega _R$, in the red detuning regime of $\Delta =\omega _B$ when the coupling laser pumping rate is fixed at $|\varepsilon _c| = 0.07\kappa$. For any fixed value of $\omega _{sw}$, the condition $\Delta =\omega _{B}$ leads to Eq. (19) which can be solved numerically. Based on the Routh-Hurwitz criteria^63^, the system is stable for just one of the solutions of $\omega _c$ where each curve in Fig. 2 has been plotted based on that specified value of $\omega _c$. Since the Bogoliubov mode of the BEC plays the role of the mechanical mode in an optomechanical system, the dips of transparency windows appearing at $\delta \approx 2\kappa$, $\delta \approx 4.14\kappa$, and $\delta \approx 7.23\kappa$ in Fig. 2a–2c correspond to $\delta =\omega _B$.

As is seen from Fig. 2a, for $\omega _{sw}=5\omega _R$ the system is in the NMS regime where the two peaks corresponding to the two eigenmodes of the system have been resolved from each other with a fairly wide transparency window between them. It is because of the fact that for that value of $\omega _{sw}$ the enhanced effective optomechanical coupling, i.e., $\zeta |a_0 |$, is greater than the threshold of *k*/2 and consequently the system is in the strong-coupling regime^64,65^, which leads to the manifestation of NMS phenomenon. However, by increasing $\omega _{sw}$ in Fig. 2b and 2c to greater values, the enhanced effective optomechanical coupling becomes smaller and consequently the two peaks come closer to each other and the transparency window becomes narrower so that for $\omega _{sw}=30\omega _R$ (Fig. 2c), where $\zeta |a_0 |$ reduces to a value lower than $\kappa /4$, the system enters the OMIT regime and the two eigenmodes overlap. In this way, one can change the regime of the system from NMS to OMIT by manipulation of the *s*-wave scattering frequency which itself is controllable through the transverse trapping frequency of the BEC^51^.

The other important phenomenon which is observable in Fig. 2a–2c is the fact that the position of the minimum of the transparency window shifts to larger frequencies as the *s*-wave scattering frequency increases. In order to explain this phenomenon, it should be noted that in bare optomechanical systems the minimum of the transparency window of OMIT appears when the frequency of the probe laser equals to the resonance frequency of the cavity^7^. Therefore, in the present hybrid optomechanical system, where the presence of the BEC causes the resonance frequency of the cavity to be shifted from $\omega _0$ to ${\tilde{\omega }}_0=\omega _0+\frac{1}{2}NU_0$, the center of transparency window of OMIT should occur at $\omega _p=\tilde{\omega _0}$. On the other hand, in the red detuned regime of $\Delta =\omega _B$ where $\omega _c\approx {\tilde{\omega }}_0-\omega _B$, the detuning between the probe and coupling lasers, i.e., $\delta =\omega _p-\omega _c$, equals to $\delta =\omega _p-{\tilde{\omega }}_0+\omega _B$. In this way, for $\omega _p={\tilde{\omega }}_0$, which is equivalent to $\delta =\omega _B$, the minimum of the transparency window appears. Finally, since $\omega _B$ is an increasing function of $\omega _{sw}$, the minimum of the transparency window shifts to larger frequencies by increasing $\omega _{sw}$.

On the other hand, the imaginary part of the reflected field amplitude has been plotted in Fig. 2$(\mathrm{a}^{\prime }$–2$\mathrm{c}^{\prime })$ versus the normalized detuning $\delta /\kappa$ for three values of $\omega _{sw}$. Here, there are two important points that should be noted. Firstly, the slope of the reflected field amplitude is negative near the resonance point of $\delta =\omega _B$ which indicates the anomalous dispersion. Secondly, the absolute value of the slope increases by increasing the *s*-wave scattering frequency so that for $\omega _{sw}=30\omega _{R}$ the imaginary part of the reflected field amplitude becomes nearly a vertical line at the resonance point (Fig. 2$\mathrm{c}^{\prime }$). In the following, it is shown how these results lead to the slow-light phenomenon in the region of transparency window.

For this purpose, we firstly introduce the reflection coefficient of the system at the probe frequency as the square of the reflected amplitude at the probe frequency as follows

20 $$\begin{aligned} R=|\varepsilon _R|^2. \end{aligned}$$

**Figure 3:**
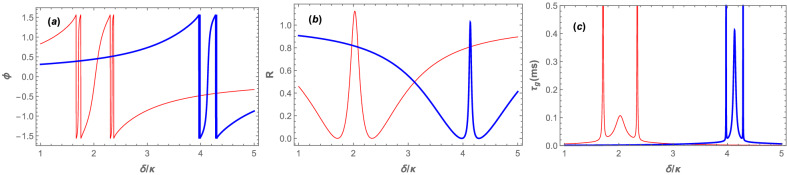
(**a**) The phase of the reflected field amplitude, (**b**) the reflection coefficient of the system, and (**c**) the group velocity delay of the output field in ms versus the normalized frequency detuning $\delta /\kappa$ for two different values of the *s*-wave scattering frequency: $\omega _{sw}=5\omega _R$ (red thin curve), $\omega _{sw}=15\omega _R$ (blue thick curve), in the red detuning regime of $\Delta =\omega _B$ when the coupling laser pumping rate has been fixed at $|\varepsilon _c| = 0.07\kappa$. The other parameters are the same as those in Fig. 2.

Then, in order to show how the atom-atom interaction affects the amount of group velocity slowness, in Fig. 3 we have plotted the phase of the reflected field amplitude, i.e., phase of Eq. (17) (Fig. 3a), the reflection coefficient of the system (Fig. 3b), and the group velocity delay of the output field due to the rapid phase dispersion in the vicinity of the resonance (Fig. 3c) versus the normalized detuning $\delta /\kappa$ for two different values of the *s*-wave scattering frequencies: $\omega _{sw}=5\omega _R$ (red thin curve) and $\omega _{sw}=15\omega _R$ (blue thick curve) in the red detuning regime of $\Delta =\omega _B$ when the coupling laser pumping rate is fixed at $|\varepsilon _c| = 0.07\kappa$.

As is seen from Fig. 3a, the phase of the reflected field amplitude has a fairly smooth variation at $\delta =\omega _B$ corresponding to the dip of transparency window where the reflection coefficient of the system is maximized as is seen from Fig. 3b, while there are two rapid variations on either side around transparency window center for each $\omega _{sw}$. It should be noted that for $\omega _{sw}=5\omega _R$ (red thin curves) the dip of transparency window occurs at $\delta \approx 2\kappa$ and for $\omega _{sw}=15\omega _R$ (blue thick curves) the dip of transparency window occurs at $\delta \approx 4.1\kappa$. Interestingly, the two rapid variations on either side of the transparency window lead to very large group velocity time delays. However, these large time delays occur at detunings where the reflected field has zero amplitude since the reflection coefficient is zero (compare Fig. 3b, 3c). Therefore, there is no physical importance for them because they cannot be detected at the output of the cavity.

Nevertheless, there is a central peak for the group velocity time delay which occurs at $\delta =\omega _B$ where the reflection coefficient is maximum. As is seen from Fig. 3c the height of central peak for $\omega _{sw}=5\omega _R$ is about $0.1 \mathrm{ms}$ while it increases to $0.4 \mathrm{ms}$ for $\omega _{sw}=15\omega _R$. Although the central peak is much weaker than the other side peaks, but the important point is that it can be physically detected at the output of the cavity because it occurs at the detuning where the reflection coefficient of the system is maximum and there is a signal with considerable amplitude. The other important point is that the central peak can be amplified by increasing the *s*-wave scattering frequency which itself is controllable through the transverse frequency of the BEC trap.

**Figure 4:**
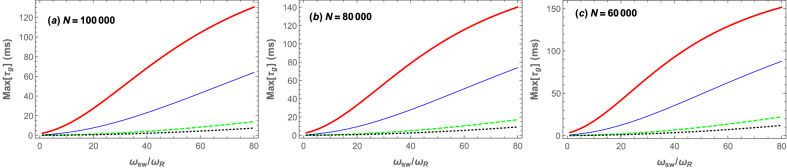
The maximum of the group velocity time delay in ms versus the normalized *s*-wave scattering frequency $\omega _{sw}/\omega _R$ for four different values of the coupling laser pumping rate: $|\varepsilon _c|=0.01\kappa$ (red thick curve), $|\varepsilon _c|=0.02\kappa$ (blue thin curve), $|\varepsilon _c|=0.05\kappa$ (green dashed curve), and $|\varepsilon _c|=0.07\kappa$ (black dotted curve) in the red detuning regime of $\Delta =\omega _B$ when the number of atoms are: (**a**) $N=100,000$, (**b**) $N=80,000$, and $N=60,000$. The other parameters are the same as those in Fig. 2.

In the following, we will show that the central peak of the group velocity time delay can be significantly increased by increasing the *s*-wave scattering frequency when the number of atoms is fixed and the cavity is driven by the coupling laser at a fixed value of pumping rate in the red detuned regime of $\Delta =\omega _B$. Then it is shown how one can also increase the central peak of $\tau _g$ by controlling the depth of the optical lattice through the coupling laser pumping rate and also by the number of the BEC atoms. For this purpose, we have plotted in Fig. 4 the maximum of the group velocity time delay corresponding to the central peak of $\tau _g$, which occurs at $\delta \approx \omega _B$ for every $\omega _{sw}$, in millisecond versus the normalized *s*-wave scattering frequency $\omega _{sw}/\omega _R$ for three different cases where the number of the BEC atoms is $N=100,000$ (Fig. 4a), $N=80,000$ (Fig. 4b), and $N=60,000$ (Fig. 4c). In each panel, the maximum of the group velocity time delay has been plotted in the red detuning regime of $\Delta =\omega _B$ for four different values of the coupling laser pumping rate: $|\varepsilon _c|=0.01\kappa$ (red thick curve), $|\varepsilon _c|=0.02\kappa$ (blue thin curve), $|\varepsilon _c|=0.05\kappa$ (green dashed curve), and $|\varepsilon _c|=0.07\kappa$ (black dotted curve)

Firstly, it should be noted that for obtaining each point of every one of curves demonstrated in Fig. 4a–4c, the cavity should be driven by the coupling laser where its frequency is tuned at a specified value of $\omega _c$ which is determined by one of the roots of Eq. (19) for every specified value of $\omega _{sw}$ where the system is stable based on the Routh–Hurwitz criteria^63^. Secondly, each point of every curve in Fig. 4a–4c corresponds to the central peak of the group velocity time delay demonstrated in Fig. 3c where the output reflected field has the maximum amplitude and therefore is physically detectable in the output of the cavity.

The important results that are obtained from Fig. 4a–4c are that one can receive a reflected slow light with a maximum amplitude in the output of the cavity so that the amount of time delay can be increased in three ways: (i) by increasing the *s*-wave scattering frequency, (ii) by decreasing the pumping rate of the coupling laser which is equivalent to decreasing the depth of the optical lattice, and (iii) by decreasing the number of the BEC atoms. Nevertheless, it should be emphasized that there are also limitations for the above-mentioned parameters. As has been mentioned earlier, the pumping rate of the cavity, from one hand, should be weak enough that the system is in the weak coupling regime where the condition $U_0 \langle a^{\dagger }a\rangle \le 10\omega _R$ is satisfied and from the other hand, it should be strong enough to generate an optical lattice which interacts with the BEC through an optomechanical coupling. Besides, the *s*-wave scattering frequency is preferable to be below $100\omega _R$ in order that our effective single-mode model of BEC to be valid. Furthermore, the number of the BEC atoms should be so large that the stability conditions of the system are satisfied.

From the experimental point of view, since $\omega _{sw}$ is controllable through the transverse trapping frequency of the BEC and the optical lattice depth can be controlled by the coupling laser pumping rate, the group time delay can be controlled experimentally by transverse trapping frequency as well as the coupling laser power. Based on the above-mentioned results which predict a group time delay of order 150 millisecond in the present setup whose cavity length is $178\,\upmu \mathrm{m}$, it can be easily concluded that the group velocity of probe laser can be reduced as low as 1 mm/s which means the realization of ultraslow light. Therefore, the most challenging problem for the experimental realization of the theoretical model proposed in this paper, is the control of the *s*-wave scattering frequency. As has been shown previously^28^, there is a one to one correspondence between the s-wave scattering frequency of the atoms and the splitting between the two peaks of the phase noise spectrum of the output cavity field. Using this correspondence, the *s*-wave scattering frequency can be calibrated by the transverse frequency of the BEC which is experimentally controllable. It is expected the present theoretical scheme to be realized experimentally if the above-mentioned challenge is overcome. Finally, it should be noted that the present scheme can experimentally lead to much smaller group velocities in comparison to previous EIT-based experiments^4,66,67^.

## Summary and conclusions

We have studied a hybrid optomechanical system consisting of a cigar-shaped BEC which is simultaneously pumped by a coupling and a probe laser where the coupling laser is resonant at the red sideband of the cavity effective frequency. If the pumping rate of the coupling laser is low enough, a shallow optical lattice is formed inside the cavity whose depth can be controlled by the coupling laser pumping rate. In the regime of weak optical lattice, the BEC wave function behaves as a single-mode quantum field in the Bogoliubov approximation which is coupled with the radiation pressure of the optical lattice and plays the role of the vibrational mode of a moving mirror in an optomechanical system.

Firstly, we have investigated the phenomenon of OMIT in such a hybrid optomechanical system and have shown how the system can switch from the NMS to the OMIT regime by increasing the *s*-wave scattering frequency of atomic collisions when the cavity is pumped at a fixed rate. Then, we have shown that an ultraslow light with a time delay as high as 150 ms, which corresponds to a group velocity as low as 1 mm/s, is achievable by decreasing the optical lattice depth through decreasing the pumping rate of the cavity and also by increasing the strength of atom-atom interaction through the transverse trapping frequency of the BEC and controlling the number of atoms of the BEC.

Furthermore, by studying the behavior of the phase of the cavity output field as well as the reflection coefficient of the cavity, we have also explicitly shown that the mentioned time delay occurs in the frequency region of coupling-probe detuning where the reflection coefficient of the cavity is maximum and therefore an ultraslow light with a considerable amplitude is physically observable in the output field of the cavity. Besides, We have also shown that there are two other ultraslow lights with larger time delays. However, they are not observable in the output of the cavity because they occur at frequencies that the reflection coefficient of the system is zero.
